# Surname lists to identify South Asian and Chinese ethnicity from secondary data in Ontario, Canada: a validation study

**DOI:** 10.1186/1471-2288-10-42

**Published:** 2010-05-15

**Authors:** Baiju R Shah, Maria Chiu, Shubarna Amin, Meera Ramani, Sharon Sadry, Jack V Tu

**Affiliations:** 1 Institute for Clinical Evaluative Sciences, 2075 Bayview Avenue, Toronto, Ontario, M4N 3M5, Canada; 2 University of Toronto, Toronto, Ontario, Canada; 3 Department of Medicine, Sunnybrook Health Sciences Centre, 2075 Bayview Avenue, Toronto, Ontario, M4N 3M5, Canada

## Abstract

**Background:**

Surname lists are useful for identifying cohorts of ethnic minority patients from secondary data sources. This study sought to develop and validate lists to identify people of South Asian and Chinese origin.

**Methods:**

Comprehensive lists of South Asian and Chinese surnames were reviewed to identify those that uniquely belonged to the ethnic minority group. Surnames that were common in other populations, communities or ethnic groups were specifically excluded. These surname lists were applied to the Registered Persons Database, a registry of the health card numbers assigned to all residents of the Canadian province of Ontario, so that all residents were assigned to South Asian ethnicity, Chinese ethnicity or the General Population. Ethnic assignment was validated against self-identified ethnicity through linkage with responses to the Canadian Community Health Survey.

**Results:**

The final surname lists included 9,950 South Asian surnames and 1,133 Chinese surnames. All 16,688,384 current and former residents of Ontario were assigned to South Asian ethnicity, Chinese ethnicity or the General Population based on their surnames. Among 69,859 respondents to the Canadian Community Health Survey, both lists performed extremely well when compared against self-identified ethnicity: positive predictive value was 89.3% for the South Asian list, and 91.9% for the Chinese list. Because surnames shared with other ethnic groups were deliberately excluded from the lists, sensitivity was lower (50.4% and 80.2%, respectively).

**Conclusions:**

These surname lists can be used to identify cohorts of people with South Asian and Chinese origins from secondary data sources with a high degree of accuracy. These cohorts could then be used in epidemiologic and health service research studies of populations with South Asian and Chinese origins.

## Background

Because many secondary data sources used in health research do not include information on race or ethnicity, surnames are often used as a proxy when studying health care for ethnic populations. In the United States, the Bureau of the Census compiled a list of "Spanish surnames" in the 1950s which has since been updated every decade [1]. These lists have been used extensively to study Hispanic populations using secondary data sources.

Canada's two largest minority populations are those with South Asian (from India, Pakistan, Bangladesh and Sri Lanka) and Chinese origins [2]. Surname lists to identify both of these population groups have been developed. To identify South Asian populations, two proprietary computerized algorithms using first and surnames were developed in the United Kingdom [3,4]. The SANGRA algorithm yielded sensitivity from 89% to 96% versus self-reported ethnicity from various data sources across England, with positive predictive value between 80% and 89% [3]. In contrast, the Nam Pehchan program had a sensitivity of 61% and a positive predictive value of 97% against self-reported ethnicity in a national sample [4]. In addition an American list of South Asian surnames was developed by Lauderdale and Kestenbaum [5]. However, it had only 38% sensitivity and 77% positive predictive value against census records. In addition, the US Census derived an Asian-Indian surname list from the 2000 Census [6], but the test characteristics of this list have not been evaluated. Sheth et al. [7] derived a list of South Asian surnames using Canadian death certificate data, by selecting people whose place of birth was South Asia or countries with large South Asian populations. They performed a telephone survey of 100 people who had surnames on the list and 100 who did not, and found that the list had a sensitivity of 96% versus self-reported ethnicity. However, this list did not specifically exclude surnames that are common to both South Asian and other populations, such as Khan, Ahmed, DeSouza or Fernandes. To identify Chinese populations, several surnames lists have been developed. In 1990, Hage et al. [8] developed a list of 145 Chinese surnames from the Melbourne, Australia telephone directory. In 1993, Choi et al. [9] published a list of over 200 Chinese surnames that was derived by finding the surnames of all people whose place of birth recorded in the Ontario vital statistics registry was China, Taiwan or Hong Kong. This list was further expanded and modified by Tjam [10] and by Quan et al. [11]. However, a limitation of most of these surname lists for both minority groups is that they are either unvalidated, or they are validated only in small settings rather than in a broad population, and so their generalizability to other contexts is uncertain.

The objective of this study was to develop and validate surname lists that could be used to identify cohorts of people with South Asian and Chinese origins from secondary data sources. The study was conducted in Ontario, Canada's largest province and also one of its most ethnically diverse. To identify such cohorts, we want to be confident that individuals identified using these surname lists truly come from the target ethnic population. Ensuring that such cohorts are representative of the entire ethnic population is secondary. Therefore, our goal was to maximize positive predictive value of the surname lists, potentially sacrificing sensitivity.

## Methods

### Development of the surname lists

To develop the South Asian surname list, we started with the previously developed Canadian list of South Asian surnames developed using death certificate data [7]. We added surnames found in community telephone directories and in an encyclopedia of surnames published by the Indian government [12]. Each name was then reviewed by at least two researchers with South Asian origins. Surnames were excluded if they were not felt to be uniquely South Asian (i.e., if the surname was common also in other populations, communities or ethnic groups). If there was disagreement between the researchers about whether or not to exclude a surname, it was reviewed by a panel of five researchers with South Asian origins until a consensus decision was reached. The final list included all surnames that were believed, by consensus, to be uniquely South Asian.

Although a comprehensive list of Chinese surnames has been previously published and validated, we noted that this list included some names that were not uniquely Chinese (e.g., Diep, Jain, Kang and Sen) [11]. As such, we were concerned about the positive predictive value of identifying cohorts of patients with Chinese origin using this list. Therefore, we repeated a similar consensus process for the surnames from that list, to create a final list of surnames believed, by consensus, to be uniquely Chinese.

### Administrative data sources

The Institute for Clinical Evaluative Sciences (ICES) is a health services research organization funded in part by the Ontario Ministry of Health and Long-Term Care (MOH) to conduct analyses of provincial health care administrative databases for policy-relevant and scientific research. One of these administrative data sources is the Registered Persons Database (RPDB), which is a registry of the assigned health card numbers for all current and former residents of the province of Ontario (current population = 13 million). The nominal file received from the MOH includes patients' surnames. In the data sharing agreement between the MOH and ICES, only three named individuals have access to this nominal file. They anonymize it prior to its release for general analytical use by removing the names and by encrypting the health card numbers. Because this encryption uses a reproducible algorithm common to all of the administrative data sources, individuals can be linked between databases via this unique encrypted number.

**Figure 1:**
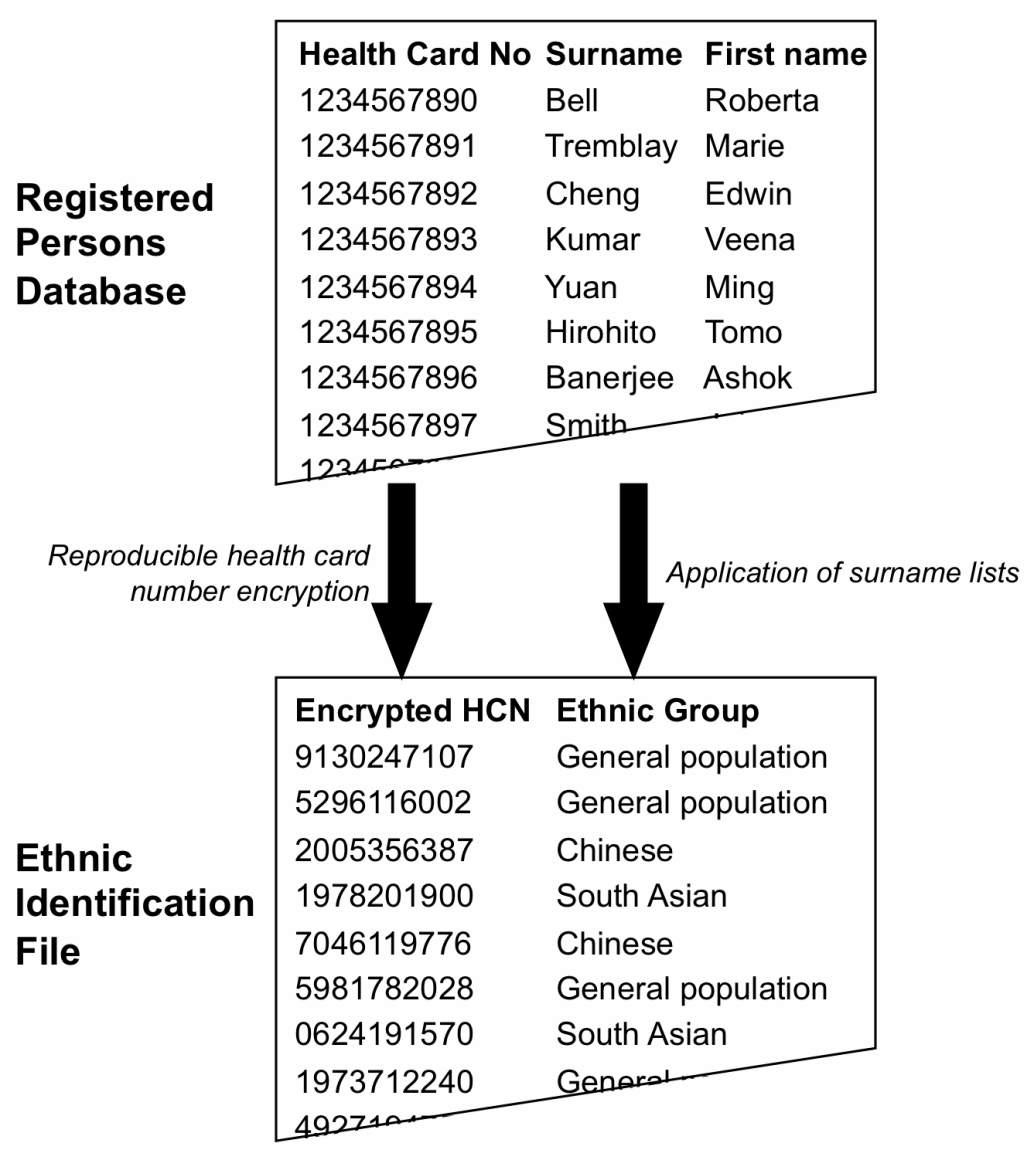
**The derivation of the surname-derived ethnic identification file from the Registered Persons Database**.

The surnames lists were applied to the nominal RPDB file to create an ethnic identification file. All Ontario residents whose surnames were on the South Asian list were assigned to South Asian ethnicity; all residents whose surnames were on the Chinese list were assigned to Chinese ethnicity; all others were assigned to the General Population group. The final surname-derived ethnic identification file included both the encrypted health card number and the ethnicity assignment for all Ontario residents. (See Figure 1.)

### Validation

We validated the surname lists against self-reported ethnicity from the Canadian Community Health Survey (CCHS). The CCHS is a recurring cross-sectional national telephone survey conducted by Statistics Canada, targeting household residents aged 12 or older, excluding those living on Indian Reserves, Canadian Forces Bases, institutions and some remote areas. Prior to 2007, the survey operated on a two-year collection cycle, with the first year examining general population health in a large sample, and the second year focusing on specific health topics in a smaller sample. Each respondent in each survey is assigned a person-level weight, so that the weight corresponds to the number of people in the entire population that are represented by that respondent, and the sum of all weights in one year's survey equals the population of Canada [13]. Three sampling frames are used for the survey, and the final weight assigned to an individual is integrated from the weights independently assigned from each sampling frame. Weights are also calibrated to account for other potential biases, including non-response and oversampling of households with multiple telephone lines. The use of the weights in analyses of these data is required to ensure findings are representative of the population, and not just of survey sample [13]. Although ethnicity is not specifically included in the derivation of respondents' weights, participants are selected at random from the population, so there is no reason to suspect that the weightings would systematically under- or over-represent any ethnic group, particularly since the survey is administered in over 20 languages. The CCHS data set does not include respondents' surnames, but respondents did give permission to have their survey responses linked with health administrative data sources via their health card number. This was anonymized using the usual encryption algorithm at ICES, so that the CCHS data can be linked with the other administrative data sources, including the RPDB.

The gold standard was self-reported ethnicity from the CCHS. Two CCHS questions, which were routinely collected as part of the demographic profile of survey respondents, were used. These were "To what ethnic groups did your ancestors belong?" and "People living in Canada come from many different cultural and racial backgrounds. Are you...?" Multiple responses were permitted for each question. Those respondents who had a single response of "South Asian" to either question were assigned to South Asian ethnicity; those who had a single response of "Chinese" to either question were assigned to Chinese ethnicity; and all others were assigned to the General Population.

We studied all adult respondents to any of the 2001, 2002 or 2003 cycles of the CCHS. For each respondent, the gold-standard ethnicity was established from their self-reported responses to the survey, while their surname-derived ethnicity was derived through linkage of their encrypted health card number from the CCHS dataset with their ethnicity assignment in the surname-derived ethnic identification file. Validity of the South Asian surname list was determined by measuring sensitivity (the proportion of people self-identified as South Asian who were detected as such by the surname list), specificity (the proportion of people self-identified as not being South Asian who were detected as such by the surname list), positive predictive value (the proportion of those detected by the surname list as South Asian who self-identified as such) and negative predictive value (the proportion of those detected by the surname list as not being South Asian who self-identified as such). Similar calculations were made to validate the Chinese surname list. In these calculations, each respondent was weighted by his or her person-level weight (divided by 3, because we were combining respondents from three survey years). We compared the previously-published Chinese surname list [11] with our Chinese surname list by comparing surname-derived ethnicity using both lists against the same gold standard. Finally, we compared the positive predictive value of each of our surname lists stratified by sex, age and immigration status.

### Ethics

The data for the study are not publicly available, but were provided to ICES by the MOH under a research agreement. ICES is permitted to hold, link and analyze these data for research purposes as a named "prescribed entity" in Ontario's health information privacy law, the *Health Information Protection Act *[14]. The study was approved by the institutional review board of Sunnybrook Health Sciences Centre.

## Results

The initial list of potentially South Asian surnames included 13,949 names to be screened. Of them, 9,950 were selected by consensus for the final list of uniquely South Asian surnames. From the initial 1,185 Chinese surnames, 1,133 were selected for the final list. Each surname list was linked with the nominal RPDB, and all 16,688,384 current and former residents of Ontario were assigned to South Asian ethnicity (500,807), Chinese ethnicity (808,670) or the General Population (15,378,907). The 200 most common surnames from each group are presented in Table 1.

**Table 1:** The 200 most common surnames from the South Asian and Chinese surname lists, and from the general population in Ontario.

South Asian		Chinese		General population	
**Name**	**N**	**Name**	**N**	**Name**	**N**

Patel	35,984	Wong	34,567	Smith	91,575

Singh	31,820	Chan	32,692	Brown	57,222

Sharma	10,216	Li	27,608	Lee	49,898

Kaur	7,462	Chen	25,618	Wilson	43,803

Persaud	6,982	Wang	22,548	Martin	38,878

Sandhu	6,229	Liu	18,784	Taylor	35,746

Grewal	6,044	Zhang	18,003	Campbell	34,551

Sidhu	5,862	Lam	15,910	Williams	34,104

Dhaliwal	5,209	Leung	13,696	Thompson	33,810

Dhillon	4,847	Ho	12,830	Jones	32,644

Agarwal, Aggarwal, Ahluwalia, Ahuja, Akhtar, Akhter, Akram, Anand, Arora, Arumugam, Atwal, Aujla, Aulakh, Bains, Bajwa, Baksh, Balachandran, Balasingam, Balasubramaniam, Banerjee, Bansal, Banwait, Bedi, Begum, Beharry, Bhalla, Bhandari, Bhardwaj, Bhatia, Bhatt, Bhatti, Bhavsar, Bhogal, Bhullar, Boodram, Boparai, Brar, Chadha, Chahal, Chand, Chandra, Chaudhary, Chaudhry, Chauhan, Chawla, Cheema, Chohan, Chopra, Choudhry, Choudhury, Chowdhury, Das, Dass, Datta, Deol, Desai, Dhami, Dhanoa, Dhindsa, Dosanjh, Gandhi, Ganesh, Garcha, Ghosh, Ghuman, Gopaul, Gupta, Heer, Hundal, Jaffer, Jafri, Jain, Jassal, Johal, Joshi, Kahlon, Kalra, Kanagaratnam, Kandasamy, Kandiah, Kanji, Kapadia, Kapoor, Karam, Karimi, Kaushal, Khaira, Khanna, Khatri, Khokhar, Kohli, Kumar, Kumarasamy, Ladha, Lakhani, Lal, Lalani, Lall, Mahabir, Mahadeo, Maharaj, Mahendran, Malhi, Malhotra, Mangat, Manji, Manoharan, Maraj, Matharu, Mathur, Mehta, Mistry, Modi, Mohan, Multani, Nadarajah, Naik, Nair, Naraine, Navaratnam, Nijjar, Panchal, Pandher, Pandya, Panesar, Pannu, Parekh, Parikh, Parmar, Parveen, Pathak, Pathan, Pathmanathan, Persad, Prajapati, Prasad, Prashad, Purewal, Puri, Rai, Raja, Rajaratnam, Rajkumar, Ram, Ramcharan, Ramkissoon, Ramnarine, Rampersad, Rampersaud, Ramroop, Randhawa, Rao, Sahota, Saini, Samra, Sangha, Sanghera, Sankar, Sehgal, Sekhon, Selvarajah, Selvaratnam, Sethi, Shanmuganathan, Shergill, Sheth, Shukla, Sinha, Sinnathamby, Sivakumar, Sivasubramaniam, Sodhi, Sohail, Sohal, Sohi, Sood, Sritharan, Subramaniam, Tharmalingam, Thind, Toor, Trahan, Trivedi, Uppal, Varghese, Verma, Virdi, Virk, Vyas, Walia		An, Au, Bai, Cai, Cao, Chang, Chao, Chau, Cheng, Cheong, Cheung, Chiang, Chin, Ching, Chiu, Cho, Chong, Chou, Chow, Choy, Chu, Chua, Chui, Chun, Chung, Cui, Dai, Deng, Ding, Dong, Du, Duong, Eng, Fan, Fang, Feng, Fok, Fong, Fu, Fung, Gao, Gong, Gu, Guan, Guo, Ha, Han, He, Hong, Hou, Hsu, Hu, Hua, Huang, Hui, Hum, Hung, Hwang, Ing, Ip, Jang, Ji, Jia, Jiang, Jin, Kam, Kan, Ko, Kong, Koo, Ku, Kung, Kuo, Kwan, Kwok, Kwon, Kwong, La, Lai, Lao, Lau, Lei, Leong, Liang, Liao, Lin, Ling, Lo, Lu, Lui, Luk, Lum, Luo, Luong, Ma, Mah, Mai, Mak, Man, Mao, Mei, Meng, Mian, Mo, Mok, Monk, Ng, Ngai, Ong, Ou, Pan, Pang, Peng, Phung, Poon, Qi, Qian, Qin, Qiu, Quan, Ren, Seto, Shao, Shen, Shi, Shum, Sin, Situ, Siu, So, Song, Su, Sun, Sung, Szeto, Ta, Tai, Tam, Tan, Tang, Tao, Tian, To, Tom, Tong, Tsai, Tsang, Tse, Tsui, Tu, Tung, Wan, Wei, Wen, Wing, Woo, Wu, Xia, Xiao, Xie, Xu, Xue, Yan, Yang, Yao, Yap, Yau, Ye, Yee, Yeh, Yeung, Yi, Yim, Yin, Yip, Yiu, Yong, Yoon, Yu, Yuan, Yue, Yuen, Yung, Zeng, Zhao, Zheng, Zhong, Zhou, Zhu, Zou		Adams, Ahmed, Alexander, Ali, Allen, Anderson, Andrews, Armstrong, Bailey, Baker, Barnes, Bélanger, Bell, Bennett, Black, Boyd, Bradley, Brooks, Burke, Burns, Butler, Cameron, Carter, Chapman, Choi, Clark, Clarke, Cole, Collins, Cook, Cooper, Cox, Craig, Crawford, Cunningham, Da Silva, Davidson, Davies, Davis, Dawson, Dixon, Douglas, Doyle, Duncan, Dunn, Edwards, Elliott, Ellis, Evans, Ferguson, Fernandes, Ferreira, Fisher, Fleming, Ford, Foster, Fox, Francis, Fraser, Gagnon, Garcia, Gauthier, George, Gibson, Gill, Gordon, Graham, Grant, Gray, Green, Hall, Hamilton, Harris, Harrison, Hart, Harvey, Hassan, Hayes, Henderson, Henry, Hill, Holmes, Howard, Hughes, Hunt, Hunter, Huynh, Jackson, James, Johnson, Johnston, Kelly, Kennedy, Kerr, Khan, Kim, King, Knight, Lalonde, Lawrence, Le, Leblanc, Lewis, Little, Macdonald, Mackenzie, Maclean, Macleod, Mann, Marshall, Mason, Matthews, McDonald, McIntyre, McKay, McKenzie, McLean, McLeod, Miller, Mills, Mitchell, Mohamed, Moore, Morgan, Morin, Morris, Morrison, Murphy, Murray, Nelson, Nguyen, O'Brien, Palmer, Park, Parker, Parsons, Patterson, Paul, Payne, Pereira, Perry, Peters, Phillips, Porter, Powell, Price, Reid, Reynolds, Richards, Richardson, Roberts, Robertson, Robinson, Rogers, Rose, Ross, Roy, Russell, Ryan, Santos, Saunders, Scott, Seguin, Shah, Shaw, Silva, Simpson, Spencer, Stevens, Stevenson, Stewart, Sullivan, Sutherland, Thomas, Thomson, Tran, Tremblay, Turner, Walker, Wallace, Walsh, Ward, Warren, Watson, White, Williamson, Wood, Woods, Wright, Young	

There were 69,859 CCHS respondents who were included in this validation study, of whom 1,400 self-identified as South Asian (5.4% of the weighted population), and 1,129 self-identified as Chinese (4.0% of the weighted population). The baseline characteristics of the population are shown in Table 2. The sensitivity, specificity and positive and negative predictive values of the South Asian and Chinese surnames lists are shown in Table 3. Both lists perform extremely well, with approximately 90% positive predictive value. The sensitivity of the South Asian list was low, as expected, since the many surnames common for but not unique to South Asians were excluded from the final list. Compared to the previously published Chinese list, our modified Chinese surname list had slightly better positive predictive value with the sacrifice of a small amount of sensitivity.

**Table 2:** Unweighted and weighted baseline characteristics of the study population

Characteristic	South Asian	Chinese	General population
*Unweighted*			

N	1,400	1,129	67,330

Surname-derived ethnicity			
South Asian	654	4	129
Chinese	9	899	139
General population	737	226	67,062

*Weighted*			

Proportion of the population	5.4%	4.0%	90.6%

Sex			
Male	54.7%	52.5%	48.4%
Female	45.3%	47.5%	51.6%

Age			
44 or younger	64.4%	62.6%	52.0%
45 to 64	29.2%	27.6%	31.4%
65 or older	6.4%	9.8%	16.6%

Immigration status			
Born in Canada	7.0%	9.6%	73.7%
Immigrant ≤10 years	47.2%	38.1%	6.0%
Immigrant 11 to 20 years	25.9%	28.3%	4.8%
Immigrant > 20 years	19.9%	24.0%	15.5%

**Table 3:** Test characteristics of the South Asian and Chinese surname lists compared against self-reported ethnicity

Surname list	Sensitivity (%)	Specificity (%)	Positive predictive value (%)	Negative predictive value (%)
South Asian	50.4	99.7	89.3	97.2

Chinese	80.2	99.7	91.9	99.2

Previously published Chinese^9^	82.5	99.7	91.2	99.3

Table 4 shows the positive predictive value of the surname lists among patients stratified by sex, age and immigration status. The surname list was slightly less likely to accurately predict self-identified ethnicity among South Asian women, but the difference between sexes was not marked among people with Chinese origin. As expected, longer time since immigration was associated with worse positive predictive value in both ethnic groups. Both lists performed poorly among those individuals who were Canadian-born.

**Table 4:** Positive predictive value (%) of the South Asian and Chinese surname lists compared against self-reported ethnicity, stratified by sex, age and immigration status.

Characteristic	South Asian	Chinese
Overall	89.3	91.9

Sex		
Male	91.4	92.1
Female	86.5	91.7

Age		
44 or younger	89.2	92.6
45 to 64	88.0	90.0
65 or older	94.6	93.0

Immigration status		
Born in Canada	58.7	77.8
Immigrant ≤10 years	95.2	95.9
Immigrant 11 to 20 years	89.6	94.1
Immigrant > 20 years	88.6	88.1

## Discussion

We have developed surname lists that can accurately identify people with self-reported South Asian or Chinese origins through secondary data sources. Despite the relatively low prevalence of minorities in the total population, both lists had excellent positive predictive value [15], so they can be used to confidently assemble ethnic minority cohorts for epidemiologic or health services research studies of people with South Asian or Chinese origins. However, the South Asian list had, as expected, low sensitivity. Therefore, it cannot be used, for example, to determine the proportion of a disease cohort that has South Asian origins. The Chinese list had a slightly better positive predictive value than the previously published list on which is was based [11], and also retained good sensitivity.

The surname lists showed markedly poorer positive predictive value for people who were Canadian-born. This most likely reflects people from other backgrounds who have adopted an ethnic surname through marriage, or to people whose parents were from different ethnic backgrounds. (However, it is noteworthy that people of South Asians and Chinese origins are the least likely in Canada to report being married to someone from outside their ethnic group [2].) It may also indicate that Canadian-born minorities may not self-identify with their ethnic group. Hence, although the surname lists are useful in the overall population, they may be less valid for use in this specific subgroup. In the future, as more people adopt surnames from other ethnic groups and as more people with multiple ethnic origins are born, the usefulness of surname lists to identify ethnic populations from secondary data may decline.

Identification of Chinese surnames is relatively straightforward, as about 95% of the Chinese population is Han Chinese. There are only a few hundred different common surnames in Chinese script [16], and a limited number of Westernized spellings of each name. In contrast, the South Asian population includes a large number of language, religious and cultural groups, each with a multitude of common surnames. Hence, our original list of South Asian surnames to screen was more than 10 times longer than the list of Chinese surnames. Because we sought to maximize positive predictive value by limiting our final list to include only those names that were uniquely South Asian, the final list includes mostly Hindu, Sikh and Sri Lankan surnames. Names used by South Asian Muslims or Christians are shared with people from those faiths in other parts of the world, and indeed may be *more *common in people with other ethnic backgrounds (e.g., Arab, Persian or Portuguese). Although including these names could have improved the sensitivity of our surname list, their inclusion would have led to an unacceptably dramatic drop in positive predictive value, and would have rendered the surname list invalid for our purposes. Other South Asian name algorithms have included such names to maximize sensitivity, but this came at a cost of very poor positive predictive value [17].

There are limitations to this methodology. As noted above, the included surnames on the South Asian list are mostly Hindu, Sikh and Sri Lankan names while surnames from other religious or cultural groups are excluded. Since there is evidence of biological and behavioural heterogeneity between South Asian populations [18-21], any cohorts built using the surname list may therefore systematically exclude people with different biological or clinical risks. The same is true for the Chinese list, although the Han population does make up an overwhelming majority of the Chinese population. In addition, there are limitations to the gold standard. We restricted our gold-standard definition to questions about ancestry and cultural/racial background. We did not use survey questions about place of birth or first language as these may not be specific nor sensitive for ethnicity. There may have been confusion about the meaning or intent of the survey questions, which might have led to incorrect responses. In particular, terms like "South Asian" (versus "Southeast Asian") or "Indian" (versus "Aboriginal") may have been confused by both respondents and interviewers. Finally, generalizing surname lists to populations outside of the areas in which they were derived is problematic, because of differing immigration patterns to different parts of the world. This phenomenon was demonstrated with the Nam Pehchan algorithm in the United Kingdom, which had a sensitivity of over 94% when evaluated for individuals in Bradford where it was developed, but only 61% when evaluated for individuals from across England [4]. Therefore, our surname list would likely not have the same performance in other jurisdictions.

## Conclusions

In conclusion, these surname lists can be used to accurately identify cohorts of people with South Asian and Chinese origins from secondary data sources, although these cohorts would, by necessity, not always be comprehensive or representative of the entire South Asian or Chinese population.

## Abbreviations

CCHS: Canadian Community Health Survey; ICES: Institute for Clinical Evaluative Sciences; RPDB: Registered Persons Database.

## Competing interests

The authors declare that they have no competing interests.

## Authors' contributions

BRS conceived of the study, participated in its design and in the surname review, performed the statistical analyses, and drafted the manuscript. MC participated in the design of the study and the surname review. SA, MR and SS all participated in the surname review. JVT participated in the design of the study. All authors revised the manuscript critically for intellectual content, and all have read and approved the final version.

## Pre-publication history

The pre-publication history for this paper can be accessed here:

http://www.biomedcentral.com/1471-2288/10/42/prepub
